# TRPV1 channel inhibition contributes to the antinociceptive effects of *Croton macrostachyus* extract in mice

**DOI:** 10.1186/s12906-015-0816-z

**Published:** 2015-08-25

**Authors:** Télesphore Benoît Nguelefack, Rafael Cypriano Dutra, Ana Flavia Paszcuk, Edinéia Lemos de Andrade, João Batista Calixto

**Affiliations:** Laboratory of Animal Physiology and Phytopharmacology, Department of Animal Biology, Faculty of Sciences, University of Dschang, P.O. Box 67, Dschang, Cameroon; Department of Pharmacology, Centre of Biological Sciences, Universidade Federal de Santa Catarina UFSC, Campus Universitario, Rua Ferreira Lima 82, Trindade, 88049-900 Florianopolis, SC Brazil; Laboratory of Autoimmunity and Immunopharmacology, Campus Araranguá, Universidade Federal de Santa Catarina, 88900-000 Araranguá, SC Brazil; Centre of inovation and preclinic studies (CIEnP), Av. Luiz Boiteux Piazza 1302, Cachoeira do Bom Jesus, Florianópolis, SC Brazil

**Keywords:** *Croton macrostachyus*, Neuropathic pain, Inflammatory pain, Antinociceptive, TRPV1

## Abstract

**Background:**

Previous study showed that extracts from *Croton macrostachyus* (Euphorbiaceae) exhibit analgesic effects in acute pain models. The present study evaluates the antinociceptive properties of the methanol/methylene chloride extract (MECM) of the stem bark of this plant using mice models of persistent inflammatory and neuropathic pain, and assesses its mechanism of action.

**Methods:**

MECM was tested on Complete Freund adjuvant (CFA)-induced persistent thermal and mechanical pain, neuropathic pain induced by partial sciatic nerve ligation (PSNL), prostaglandin E_2_ (PGE_2_)-induced acute mechanical hyperalgesia, as well as on nociception induced by capsaicin in mice. Mechanical hyperalgesia was assessed using von Frey hair in awake mice. The mechanism of action of MECM was evaluated by using glibenclamide on PGE_2_-induced hyperalgesia or rimonabant on capsaicin-induced pain.

**Results:**

MECM administered orally at the doses of 250 and 500 mg/kg, induced long lasting and significant antihyperalgesic effects on CFA-inflammatory and PSNL-induced neuropathic pain. MECM significantly reduced the mechanical hyperalgesia induced by PGE_2_ either when administered preventively or therapeutically. MECM also significantly and time dependently inhibited the capsaicin-induced nociception. These effects were not affected by glibenclamide or by rimonabant.

**Conclusions:**

The present results demonstrate that the oral administration of MECM to mice resulted in long lasting antihyperalgesic activity in inflammatory and neuropathic pain as well as in acute and persistent pain. The mechanism underlying the long lasting MECM antihyperalgesic effect is currently unknown, but might be mediated, at least partially, through the modulation of TRPV1 receptors.

## Background

Pain is a subjective and unpleasant experience willing to protect the organism against harmful stimuli. The unpleasant sensation in acute pain and the useless long-term emotional experience of suffering in chronic pain have turn pain to become an entire disease that needs to be alleviated as early as possible. In addition, unremitting pain is frequently associated with anxiety, depression, loss of independence, and interference with work and relationships. The anxiety and the emotion resulting from pain, reciprocally strengthen the pain and thereby, maintain a vicious circle that worse the patient condition. Furthermore, chronic neuropathic pain can acquire with time an important inflammatory component [1, 2] which is thought to account for neuronal plasticity associated to pain. Indeed, it has been shown that in both neuropathic and inflammatory pain conditions various neuronal cells proteins are up regulated, including TRPV1 receptors [3]. Apart from TRPV1 antagonists, the activity of TRPV1 channels can be modulated by cannabinoid receptors which are collocated in the same nerve endings or by ATP sensitive potassium channels. These cell structures are potential targets for pain management.

For many years, research has been focused on seeking medicine that can conveniently address chronic pain. But, still more than 40 to 50 % of patients in routine practice settings fail to achieve adequate relief especially those suffering from neuropathic pain [4]. Thus, pain, especially chronic pain, resulting from inflamed tissues or directly from nerve injury are now considered to be a public health problem of major proportions. Hence, there is a great need for the development of new, safe and better analgesic drugs for the alleviation and control of acute and chronic, inflammatory and neuropathic pain. Medicinal plants are considered as an important source of active molecules of pharmacological interest, given their efficiency proved against many diseases and the fact that more than 50 % of the actual manufactured drugs are from plant or derivatives of plant compounds [5, 6].

*Croton macrostachyus* (Hochst) pipe cleaner ex and Galinier, is a plant used in African folk medicine to treat different ailments, including pain and inflammation [7, 8]. Indeed, previous studies have shown that aqueous and organic extracts from the stem bark of *C. macrostachyus* given orally, possess antinociceptive and anti-inflammatory activities in distinct acute animal models [9]. The present study aimed at providing additional insight about the antinociceptive effects of the methanol/methylene chloride extract of the stem bark of *C. macrostachyus* in different models of chronic inflammatory and neuropathic pain and to evaluate its preliminary mechanisms of action.

## Methods

### Plant collection and extraction

Plant material was harvested in Bagangté (West, Cameroon) and identified at the Cameroon National Herbarium in comparison to the existing Voucher specimen (5696/SRF/CAM). The stem bark was sun dried and pulverized. Six kilograms of *C. macrostachyus* powder were macerated in 10 L of a mixture of methanol/methylene chloride (CH_3_OH/CH_2_Cl_2_, 1:1 v/v) for 2 days at room temperature, filtered and concentrated at 45 and 65 °C successively, using a rotary evaporator. This process yielded 160 g of CH_3_OH/CH_2_Cl_2_ extract, which correspond to 2.67 % yield. This extract has been shown to possess alkaloids, phenols, terpernoids and flavonoids [9], including lupeol, betulin floridolide A, hardwickic acid and 12-oxo-hardwickic acid [10].

### Animals

Swiss mice (25–35 g) of both sexes, aged 6 to 8 weeks were used in the present study. Animals were obtained from the central animal house of the Department of Pharmacology from Federal University of Santa Catarina, Brazil. They were acclimatized for at least 1 week in the laboratory at a controlled temperature (22 ± 1 °C), humidity (50–80 %), under a 12-h light/dark cycle with free access to standard commercialized rodent diet and filtrated water. The number of mice used was the minimum possible to determine consistent effects of the drug treatments (See figures). All protocols were submitted and approved by the Ethical Committee for use of Animals of the Federal University of Santa Catarina (protocol no. PP00496) and conformed to the guidelines for the study of pain in awake animals established by the International Association for the Study of Pain.

## Experimentation

### Mechanical hyperalgesia behaviour assessment

Mice were acclimatized for at least 1 hour in individual transparent cages (9 × 7 × 11 cm) placed on an elevated wire mesh platforms. They were evaluated for paw withdrawal using 0.4 g von Frey hair (VFH, Stoelting, Chicago, IL, USA). Each animal was essayed 10 times and the result was expressed as percentage of response (paw withdrawal) to the number of stimulations, as previously published [11].

### Complete Freund Adjuvant (CFA) - induced mechanical hyperalgesia

Before any treatment, the baseline percentage withdrawal was measured. Then, animals were treated orally with vehicle (10 ml/kg), MECM (250 or 500 mg/kg) or gabapentin (used as positive control drug, 70 mg/kg). One hour after treatment, they were slightly restrained and received an intraplantar injection of 20 μl of CFA (100 %) and returned in wire mesh plate form. The frequency of response to von Frey hair was then evaluated at 1, 2, 4, 6, 8 and 24 h time points after CFA injection. After the 24 h hyperalgesia evaluation, animals received another dose of treatment and were further kept untreated while evaluating pain behavior until the response frequency was equal to that of control group receiving vehicle [11]. Then they received once daily treatment and were evaluated for pain sensation 24 h after each administration until 14 days post CFA. The aim of this protocol was to verify if repeated treatment could result in a long lasting analgesic effect.

### Neuropathic pain induced by sciatic nerve partial ligation

To evaluate neuropathic pain-like behavior, the procedure was similar to that described previously [12]. Mice were anaesthetized by intraperitoneal injection of chloral hydrate 7 % (0.8 ml/kg), after the measurement of the basal response to mechanical stimulation with von Frey hair 0.4 g. The right common sciatic nerve was exposed at the level of the mid-thigh, proximal to the nerve trifurcation. The exposed nerve was partially (1/3 to 1/2) ligatured using 8.0 suture needles. Care was taken to preserve epineural circulation. The tight was slowly stretched until the ispilateral hind foot elicited a brief twitch. Then the incision was sutured and treated with iodine. Five days after the surgical intervention, a second mechanical basal pain behavior was assayed to assure the effectiveness of pain installation. Then, animals were treated orally either with vehicle, MECM (500 mg/kg) or gabapentin (used as positive control drug, 70 mg/kg) and hyperalgesia was tested 1, 2, 4, 6, 8 and 24 h time points post treatment on the right hind paw. After the 24 h measurement, animals were treated twice a day (10 a.m and 6 p.m) and neuropathic pain-like behavior was evaluated the next day before and 2 h after the first administration of the day (10 a.m).

### PGE_2_-induced mechanical hyperalgesia

After assessment of the basal reaction of male mice to von Frey hair, animals were orally treated with vehicle (DMSO/Tween), MECM (500 mg/kg) or dipyrone (used as positive control drug, 120 mg/kg) and 1 hour later, they received PGE_2_ (0.1 nmol/paw) under the subplantar aponevreous. Frequency response to von Frey hair stimulation was measured 1, 2, 4, 6 and 8 h time points post PGE_2_ [13].

In another set of experiment, treatments were given orally 1 hour after PGE_2_ (therapeutic) and pain was evaluated till 8 h post PGE_2_ injection. The post treatments with MECM (500 mg/kg) or dipyrone 120 mg/kg, were evaluated in absence and in presence of glibenclamide (5 mg/kg, i.p), an inhibitor of ATP - sensitive potassium channels. The inhibitor was used to evaluate the participation of ATP-dependant potassium channel in the analgesic effects of MECM. Antagonist was administered 30 min before MECM or dipyrone.

### CFA – induced thermal hyperalgesia

Male mice latency time of reaction against infrared radiation (intensity 15 % in the paw), was measured and animals were then treated orally with vehicle (10 mL/kg), MECM (500 mg/kg p.o.) or dipyrone (120 mg/kg, p.o.). One hour after the treatment, they were intraplantarly injected with 20 μl of CFA and pain behavior was again assessed 1, 2, 4, 6, 8 and 24 h time points after CFA injection, as previously described [14].

### Capsaicin-induced spontaneous nociception

Male mice were treated with vehicle, MECM (500 mg/kg, p.o) or with capsazepine (11.3 μg/paw) and acclimated in glace funning for at least 20 min before challenged with 1.6 μg/paw of capsaicin. Pain behavior was evaluated at 1, 2, 4 or 6 h time points after MECM treatment. Different groups of animals were used at each time point. After capsaicin injection, animals were returned in individual funning and observed for the consecutive 5 min. The time the animal spent licking the injected paw was timed with chronometer and consider as indicative of pain [15]. Capsazepine used as positive control was injected *in situ* and appropriate control was conducted.

To assess whether the effect of MECM was mediated through cannabinoid CB_1_ receptors that are collocated in the same neurons with TRPV1 capsaicin sensitive receptor [16], the effect of the extract was assessed in animals previously treated with rimonabant (10 mg/kg, p.o), an inverse agonist of CB_1_ receptor. WIN 55,212-2 **(**(*R*)-(+)-[2,3-Dihydro-5-methyl-3[(4-morpholinyl)methyl] pyrrolo[1,2,3-de]-1,4-benzoxazinyl]-(1-naphthalenyl) methanone, 3 mg/kg, i.p) was used as positive control. Appropriate controls were conducted both for rimonabant and WIN 55,212-2. In the course of experiment, rimonabant was given 30 min before WIN 55,212-2 and MECM and animals were challenged with capsaicin 30 min after WIN 55,212-2 or 1 h after MECM administrations. This time schedule was chosen base on the time course effect of the extract in previous experiments as well as that of the WIN 55,212-2 and rimonabant in the literature [17].

### Drugs

Complete Freund Adjuvant (CFA), capsaicin, capsazepine, Win 55,212-2, prostaglandine E_2,_ dipyrone, gabapentin and cremophore were purchased from Sigma Chemical CO, (St. Louis, MO, USA). Glibenclamide was obtained from RBI and rimonabant from Sanofi. Gabapentin and dipyrone were prepared in saline. Plant extract and Win 55,212-2 were prepared in saline containing 5 % of DMSO and 5 % Tween 80 which was further used as vehicle in control animals. Glibenclamide was prepared in saline containing 2 % of DMSO and 2 % Tween 80. Capsaicin stock solution (10^−2^ M) was prepared by successively dissolving capsaicin in 10 % ethanol, 10 % Tween 80 and 80 % NaCl 0.9 %. The stock solution was further diluted in saline upon administration to 80 μg/ml. Stock solution of capazepine (10^−2^ M) was prepared in 20 % ethanol and further diluted upon administration to the concentration of 565 μg/ml using saline. Prostaglandine E_2_ stock solution (10^−3^ M) was prepared in 10 % ethanol and further diluted 200 folds to a final solution of 1.76 μg/ml using Saline. Rimonabant was suspended in 5 % DMSO and 0.5 % cremophore. In all cases, vehicle solutions were prepared just before experiments and and used in control groups.

### Statistical analysis

Data are expressed as mean ± standard error of the mean of 5–7 individual animals. Data were analyzed by either one way ANOVA followed by Tukey post hoc test or two way ANOVA followed by Bonferonni as post hoc test using the GraphPad Prism software (GraphPad Software Inc., San Diego, CA). *P* < 0.05 was considered significant.

## Results

### Effects of MECM on CFA – induced mechanical hyperalgesia

**Fig. 1:**
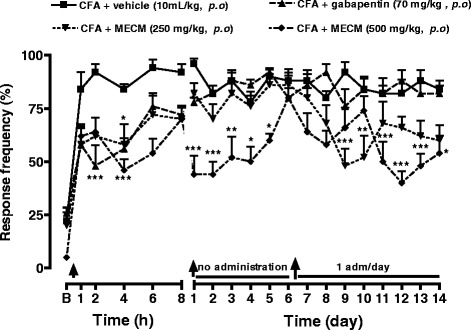
Effects of oral administration of the methanol/methylene chloride extract of the stem bark of *Croton macrostachyus* (MECM) on the mechanical hyperalgesia induced by von Frey hair (0.4 g) in CFA (20 μl/paw) inflamed mice paw. In persistent treatment, response to pain was evaluated before the treatment of the day, when given. B = basal response to mechanical stimulation. *N* = 5; ^a^ *p* < 0.05; ^b^ *p* < 0.01; ^c^ *p* < 0.001 significantly different compared to vehicle (ANOVA two way and Bonferoni as post hoc test)

CFA injection into the mice paw induced mechanical hyperalgesia when assessed by von Frey stimulation as evidenced by the significant (*p* < 0.001) increase in the baseline frequency response. This hypersensitivity was significantly reduced by previous treatment of animals with gabapentin and also with MECM. It is worth noting that following 2 day administration of MECM at the dose of 500 mg/kg, the antihyperalgesic effect of MECM was maintained for up to 5 days. The reinitiating of treatment after the animals have recovered their hypersensitivity induced a progressive and long lasting analgesic effect, the higher dose being the most effective (Fig. 1).

### Effects of MECM on mechanical hyperalgesia in mice with partial sciatic nerve ligation

**Fig. 2:**
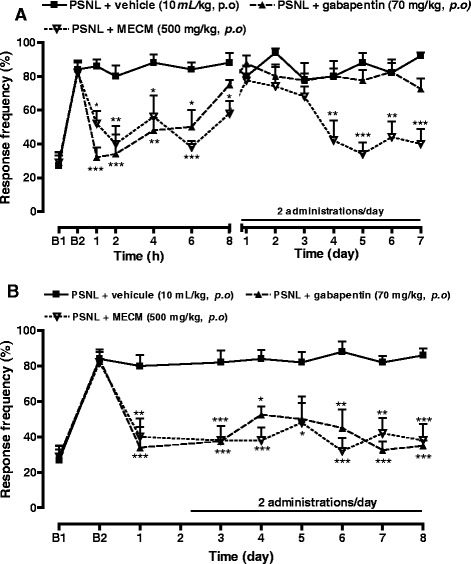
Effect of oral administration of the methanol/methylene chloride extract of the stem bark of *Croton macrostachyus* (MECM) on the mechanical hyperalgesia assessed with von Frey hair (0.4 g) in mice paw after sciatic nerve partial ligation (PSNL). In panel (**a**), response to pain was evaluated before the first administration of the day while in panel (**b**) it was evaluated 2 h after the first administration of the day. *N* = 5; ^a^ *p* < 0.05; ^b^ *p* < 0.01; ^c^ *p* < 0.001 significantly different compared to vehicle at each time point. B1 = basal response to mechanical stimulation before operation, B2 = basal response to mechanical stimulation 5 days after operation

Five days after surgery, a significant increase (*p* < 0.001) on the baseline frequency response was observed in all animals. In the acute treatment (Fig. 2a), oral administration of MECM (500 mg/kg, p.o.) significantly reduced the mechanical hyperalgesia caused by partial sciatic nerve ligation. The effect of the extract as that of gabapentin, started 1 h after administration. The antinociceptive effects of the two substances were maintained for up to 8 h; but 24 h after the administration, the sensitivity of treated animals was equal to that of control group. When given twice a day and evaluated in the next days (Fig. 2a), gabapentin did not show any long lasting effect up to the end of the experiment while MECM progressively and significantly inhibited the mechanical hyperalgesia (Fig. 2a). In contrast, when the treatment was given twice a day and pain behavior assessed 2 h after the first treatment of the day, either extract or gabapentin, almost completely inhibited the hyperalgesia (Fig. 2b).

### Effects of MECM on PGE_2_ – induced mechanical hyperalgesia

**Fig. 3:**
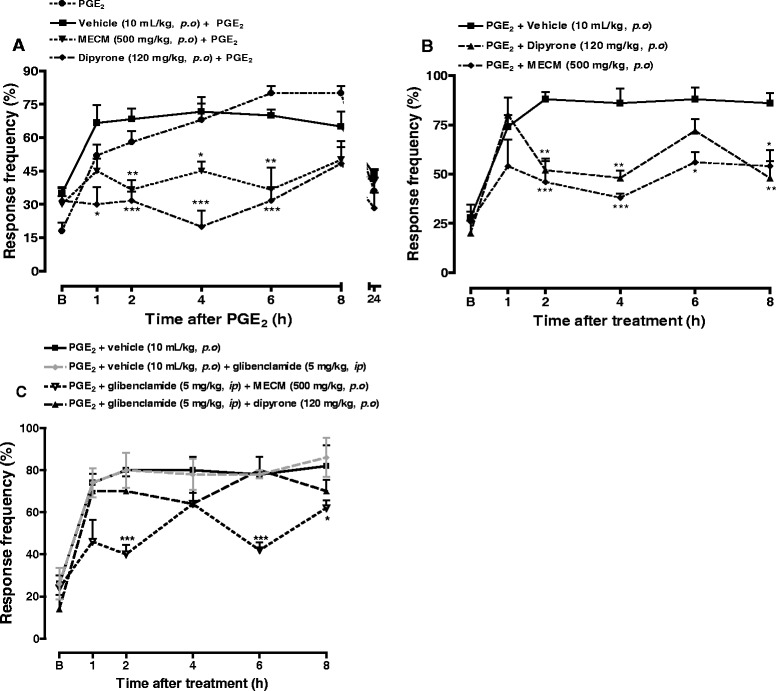
Effect of oral administration of the methanol/methylene chloride extract of the stem bark of *Croton macrostachyus* (MECM) on the mechanical hyperalgesia evaluated with von Frey hair (0.4 g) in PGE_2_ (0.1 nmol/paw) inflamed mice paw. In panel (**a**), animals were treated 1 hour before PGE_2_ injection and pain response was evaluated before treatment and after PGE_2_ injection. In panel (**b**) and (**c**), treatments were given orally 1 hour after PGE_2_ and response to pain was evaluated before PGE_2_ injection and after treatment. *N* = 5; ^a^ *p* < 0.05; ^b^ *p* < 0.01; ^c^ *p* < 0.001 significantly different compared to vehicle. B = basal response to mechanical stimulation

Intraplantar injection of PGE_2_ resulted in a significant (*p* < 0.01) hyperalgesia to mechanical stimulation when assessed by von Frey hair. PGE_2_–induced hyperalgesia was significantly reduced by dipyrone (120 mg/kg, p.o.) and by MECM (500 mg/kg, p.o.) when both were given as preventive treatment (Fig. 3a). As shown in Fig. 3b, both substances significantly inhibited PGE_2_-induced hyperalgesia when administered as a therapeutic scheme of treatment. However, glibenclamide significantly reduced the antihyperalgesic effect of dipyrone but failed to prevent that of MECM although an effect was observed at the 4th hour (Fig. 3c).

### Effects of MECM on CFA – induced thermal hyperalgesia

**Fig. 4:**
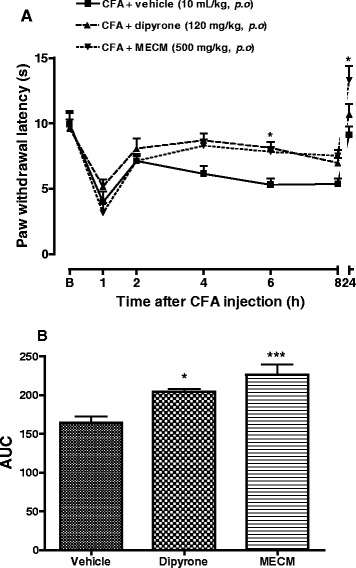
Effect of oral administration of the methanol/methylene chloride extract of the stem bark of *Croton macrostachyus* (MECM) on the thermal hyperalgesia in CFA inflamed mice paw. Treatments were given 1 h before intraplantar injection of CFA (20 μl). *N* = 6 mice per group. B = basal response to thermal stimulation. *p < 0.05, ***p < 0.001compared to control (vehicle)

CFA injected in ventral aponevreous of mice’s paw induced a significant (*p* < 0.01) thermal hyperalgesia. The reaction latency time of animals was reduced by 60 % 1 hour after CFA injection. Both dipyrone (120 mg/kg, p.o.) and MECM (500 mg/kg, p.o.) given one hour before CFA, significantly increased the latency time (Fig. 4a). The two drugs significantly reduced the sensitivity to thermal stimulation by 24.5 and 37.3 %, respectively as calculated from area under the curve (Fig. 4b).

### Effects of MECM on capsaicin-induced pain

**Fig. 5:**
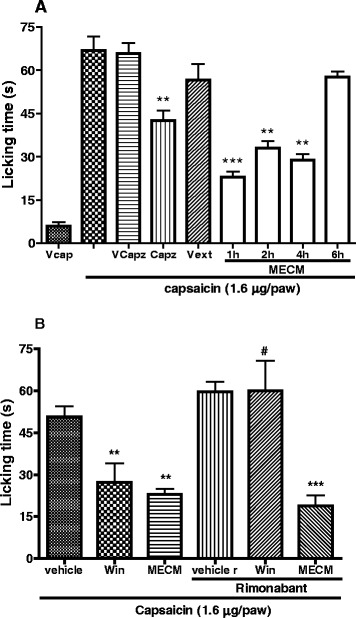
Effect of oral administration of the methanol/methylene chloride extract of the stem bark of *Croton macrostachyus* (MECM, 500 mg/kg, po) on the spontaneous pain induced by capsaicin. Panel (**a**) shows the time course effect of MECM in different groups of animals. Panel (**b**) presents the effect of rimonabant (SR 141716, 10 mg/kg, po) on the analgesic activity of MECM and WIN 55212–2 (Win, 3 mg/kg, ip) on nociception induced by capsaicin. MECM was given 1 h before intraplantar injection of capsaicin while rimonabant (10 mg/kg, p.o) was given *per os* 30 min before Win and MECM. V = vehicle, cap = capsaicin, capz = capsazepine, ext = extract. *N* = 4 (Vcap) to 7 mice per group. **p < 0.01, ***p < 0.001 significantly different compared to respective controls. # *p* < 0.01 significantly different compared to the same treatment without rimonabant

Oral administration of MECM induced a significant inhibition of pain induced by intraplantar injection of capsaicin. The extract was effective when dosed one hour after administration and its effect lasted for up to 4 h (Fig. 5a).

It is well known that capsaicin activates TRPV1 cation channels. These channels are found in the same C nerve fibers with the cannabinoid CB_1_ receptors [16]. Thus, we hypothesized that activation of these CB_1_ receptors could inhibit the capsaicin induced pain. So, the effect of WIN 55,212-2 (a CB_1_ agonist) was tested and showed antinociceptive effect on this model. To evaluate whether the effect of MECM was mediated through CB_1_ receptors, its activity as well as that of WIN 55,212-2 was evaluated in animals pretreated with rimonabant (an antagonist of cannabinoid CB_1_ receptors). Rimonabant completely reversed the antinociceptive effect of WIN 55,212-2 as expected, but failed to inhibit the effect of MECM (Fig. 5b).

## Discussion

The present study investigates the antinociceptive effects of the methanol/methylene chloride extract of *Croton macrostachyus* (MECM) in models of persistent inflammatory and neuropathic pain and explored its preliminary mechanism of action. Results demonstrated that MECM possesses orally antinociceptive property when assessed in both CFA and sciatic nerve partial ligation as well as in prostaglandin E_2_ (PGE_2_)-induced acute pain. MECM also showed significant inhibitory effect in capsaicin induced pain, an effect that was not prevented by rimonabant, a CB1 receptor reverse agonist or by glibenclamide, a ATP- dependent potassium channel inhibitor. It is important to mention, that the antinocicepetive property of MECM as demonstrated in the present paper corroborates many previous works carried out in other plants belonging to the genus Croton [18–20]. However, new experiments should be conducted to isolate and chemically characterize the active compound(s) present in the MECM.

Hyperalgesia resulting from CFA injection in animals’ paw is a well known and characterized model of pain involving both the peripheral and the central nervous system. Indeed, CFA injection induces the release of histamine and serotonin from mast cells through the degranulation process and activates inflammatory cells to release cytokines (TNF-α, IL-1β, IL-6) and chemokines (MCP-1, KC, IP-10) both at the periphery and at the central nervous system [21, 22] Cytokines are responsible for the hypersensitisation of peripheral and central neurons, a phenomenon that includes the appearance of inducible proteins, up-regulation and activation of various membrane and intracellular pathways. It is now well recognized that CFA up regulates and potentiates voltage-gated sodium channels, GluR1 and TRPV1 receptors [23–25] which participate in the maintenance of persistent mechanical and thermal inflammatory pain. Herein, our data demonstrated that MECM significantly reduced the mechanical hyperalgesia induced by intraplantar injection of CFA. Moreover, the antinociceptive effect of MECM was maintained for up to 5 days after two administrations in the first 24 h. These results suggest that MECM might interfere with the acute pain sensitization as well as the over expression of pain regulatory cytokines and proteins. In other to verify this hypothesis, animal were left untreated until the sensitivity returns to the control point, then the treatment was reinitiated. It was observed that the MECM administered once a day, produced antihyperalgesic effect that reappears after the third day, showing a progressive effect. It can then be thought that MECM do not inhibit the installation of pain but rather interact with signaling pathways that induce mechanical pain in inflammatory conditions. Another possibility is that MECM may be able to reduce the well-established chronic pain. This hypothesis was evaluated by testing MECM on a well installed neuropathic pain induced by PSNL. MECM significantly reduced the PSNL neuropathic pain. On this model of pain generally known as resistant to treatment, MECM almost completely inhibited the hyperalgesia 2 hours after its oral administration. Relevantly, a long lasting effect of MECM was observed in this model from the fourth day after the initiation of the treatment. This effect was observed during the 14 days of treatment. These results suggest that MECM may have cumulative effects or may interfere with slow regulatory processes such as proteins and mediators expression. Further experiments are necessary to prove this hypothesis.

Pro-inflammatory substances and proteins released or up regulated by CFA and implicated in the mechanical hyperalgesia including inducible nitric oxide synthase and cyclooxygenase-2 [26]. Subsequent upregulation of PGE_2_ production but also of PGE_2_ receptors have been demonstrated both in inflammatory pain and in partial sciatic nerve ligation [27]. In order to determine whether MECM interferes with the PGE_2_ pathway, it was tested as preventive and therapeutic scheme of treatment on the mechanical hyperalgesia induced by this inflammatory mediator. In both cases, MECM exhibited a significant antihyperalgesic effect. As MECM was able to reduce pain when given after PGE_2_ injection, it can be conclude that this extract inhibits the downstream PGE_2_ pathway or interact with ion channels that are involve in nerve action potential or nerve conductivity.

Opening of the ATP-sensitive potassium channels (K-_ATP_) have been shown as the end mechanism of the peripheral effect of many analgesics such as morphine [28], dypirone [29], diclofenac [30], xylazine [31], sildenafil [32] and even plant derivatives [11]. In order to determine whether or not this pathway is involved in the activity of MECM, its antinociceptive effect was evaluated in animals previously treated with glibenclamide, an inhibitor of K-_ATP_ channels. Glibenclamide failed to significantly antagonize the analgesic effect of MECM, implying that its antinociceptive action is likely unrelated with the activation of the K-_ATP_ channels.

The linkage of PGE_2_ to its cellular receptors leads to the activation of protein kinase C (PKC) and protein kinase A (PKA) resulting in the release of sympathetic amines and the sympathetic pain [33] or in the activation and phosphorylation of numbers of ion channels [34], respectively. One of the channels target by PKA and PKC activation is TRPV1 [35]. This channel is one of the main receptors involve in the sensitization and the maintenance of mechanical hyperalgesia in both inflammatory and neuropathic pain [36]. It can be activated by noxious heat, low pH or chemicals [37]. For this reason, MECM was tested on thermal hyperalgesia induced in CFA-inflamed mice, and relevantly, the extract significantly reduced the hyperalgesia, indicating a possible inhibition of TRPV1 receptor.

To further ascertain the mechanism of action of MECM, it was assesseed on pain induced by capsaicin, a natural product that specifically and directly activates TRPV1 receptor [38]. Here, MECM showed a significant antinociception effect on capsaicin-induced pain that was maintained for up to 4 h, strengthening the hypothesis that the antihyperalgesic effect of this extract is at least partially mediated by the inhibition of TRPV1 channel.

There is evidence that TRPV1 and CB1 receptors are colocalized in the same neurons both at the periphery and central nervous system and that the activation of CB1 receptors may inhibit TRPV1 receptors [16, 38]. To verify whether the inhibition of capsaicin-induced pain observed with MECM is mediate by the activation of CB1 receptor, it was tested in animals that have been previously treated with rimonabant, an antagonist of CB1 receptor. Rimonabant completely reversed the antinociceptive effect of WIN 55,212-2 (cannabinoid agonist), but failed to affect that of MECM, suggesting that the effect of this extract is not mediated through CB1 receptor.

## Conclusions

Taking all together, the present study demonstrates that methanol/methylene chloride extract of the stem bark of *Croton macrostachyus* possesses relevant oral antinociceptive effects both when assessed in acute, chronic, inflammatory and neuropathic pain. The long lasting MECM antinociceptive effect seems to be mediated, at least partially, through the modulation of TRPV1 receptors.
